# Association between Nutrition Literacy and Bangladeshi Adults’ Healthy Eating Behaviors: Evidence from the Nutrition Literacy Study 2021

**DOI:** 10.3390/healthcare10122508

**Published:** 2022-12-11

**Authors:** Md. Hasan Al Banna, Mohammad Hamiduzzaman, Satyajit Kundu, Mst. Sadia Sultana, Abdul-Aziz Seidu, Keith Brazendale, Mohammad Tazrian Abid, Tasnu Ara, M. A. Rifat, N. H. M. Rubel Mozumder, John Elvis Hagan, Md Shafiqul Islam Khan, Thomas Schack

**Affiliations:** 1 Department of Food Microbiology, Faculty of Nutrition and Food Science, Patuakhali Science and Technology University, Patuakhali 8602, Bangladesh; 2 Faculty of Health, Southern Cross University, Gold Coast, Bilinga, QLD 4225, Australia; 3 School of Public Health, Southeast University, Nanjing 210096, China; 4 Faculty of Nutrition and Food Science, Patuakhali Science and Technology University, Patuakhali 8602, Bangladesh; 5 Department of Public Health and Informatics, Jahangirnagar University, Savar, Dhaka 1342, Bangladesh; 6 Department of Population and Health, University of Cape Coast, Cape Coast PMB TF0494, Ghana; 7 College of Public Health, Medical and Veterinary Sciences, James Cook University, Douglas, QLD 4811, Australia; 8 Department of Health Sciences, University of Central Florida, Orlando, FL 32816, USA; 9 Department of Food and Nutrition, College of Home Economics, Azimpur, Dhaka 1205, Bangladesh; 10 Department of Global Public Health, Karolinska Institutet, 17177 Solna, Sweden; 11 Department of Food Science and Nutrition, Hajee Mohammad Danesh Science and Technology University, Dinajpur 5200, Bangladesh; 12 Department of Health, Physical Education & Recreation, College of Education Studies, University of Cape Coast, Cape Coast PMB TF0494, Ghana; 13 Neurocognition and Action-Biomechanics-Research Group, Faculty of Psychology and Sports Science, Bielefeld University, Postfach 10 01 31, 33501 Bielefeld, Germany

**Keywords:** nutrition literacy, healthy eating behavior, adults, Bangladesh

## Abstract

This study investigated the association between healthy eating behaviors and nutrition literacy in a sample of Bangladeshi adults. A cross-sectional survey was conducted among 400 adults from two districts of Bangladesh (Dhaka and Chattogram). Data were generated by in-person interviews using a structured questionnaire. The Nutrition Literacy Scale and National Dietary Guidelines for Bangladesh were used to assess nutrition literacy and healthy eating behaviors, respectively. Multiple linear regression models were used to observe the association. The mean score for healthy eating behavior was 21.8 (SD = 4.8, Range: 5–33) on a scale of 34. A moderate positive correlation was found between nutrition literacy and healthy eating behavior of participants (r = 0.28, *p* < 0.001). The adjusted regression model showed that a 1 unit increase in nutrition literacy reflected an increase in the healthy eating behavior score of participants by 0.22 units (β = 0.223, *p* < 0.001). Findings showed an association between nutrition literacy and eating behaviors in Bangladeshi adults. Future research could be carried out to establish a causal relationship that may help inform the necessity of educational interventions for Bangladeshi adults to assist with meeting national nutrition-related targets.

## 1. Introduction

Nutrition illiteracy, unhealthy eating practices, and malnutrition are closely linked and remain a continuous issue for all population groups in developing countries, such as Bangladesh [1,2,3]. The concept of health literacy drives dietitians, nutritionists, and researchers to construct the ‘nutrition literacy’ terminology, which could be defined as, ‘the degree to which individuals have the capacity to obtain, process, and understand nutrition information and skills needed in order to make appropriate nutrition decisions’ [2,4]. Studies regarding the association between nutrition literacy and eating behaviors represent that persons with a lack of literacy often miss reading food labels and experience difficulties in interpreting the label and estimating the required proportion of food items for a balanced diet [5,6,7,8]. Furthermore, limited nutrition literacy was found to be associated with obesogenic eating behaviors [9]. In Bangladesh, malnutrition resulted in being overweight and obese for a significant proportion of adults aged ≥18 years who lived with chronic illness [10]. Recent evidence showed that more than half of Bangladeshi adults were suffering from at least one form of malnutrition (i.e., 30.4% underweight, 18.9% overweight, and 4.6% obese) [10,11], making them more vulnerable to cardiovascular diseases and diabetes mellitus. In Bangladesh, studies on the determinants of healthy eating behaviors are limited, therefore, we aimed to investigate the association between nutrition literacy and healthy eating behaviors in a sample of adults.

The socio-behavioral risk factors of malnutrition are inadequately understood in Bangladesh, and certain objectives aimed at reducing malnutrition and related diseases had fallen short. For example, Goal 1 of the United Nations Millennium Development Goals, which is ‘eradicate extreme poverty and hunger’, was not achieved in Bangladesh [12]. On the other hand, Bangladesh is making good progress to achieve Goal 2 of the Sustainable Development Goals (SDGs), which is ‘Zero Hunger’; however, gaps were highlighted to access what is needed to meet the target of achieving food security and malnutrition eradication by 2030 [13]. 

Existing evidence reports certain socio-behavioral risk factors for malnutrition; with the social aspect focusing on poor health education and nutrition illiteracy, and the behavioral risk factors identified as unhealthy eating practices [3,14]. These risk factors are complex, connected, and imperative to understand, especially in South Asian countries such as India, Pakistan, and Bangladesh where evidence shows that many adults do not have the social or economic resources to follow healthy eating recommendations [14,15,16]. Friel, Hattersley, and Laura (2015) [17] portrayed how social norms related to food supply and consumption, daily living conditions, early childhood education, and individual beliefs and attitudes influence a person’s malnutrition in Australia. In studying these complex relationships, nutrition literacy was found to play a pivotal role in Taiwanese students’ healthy eating practices [3]. A study conducted in Turkey found that adults’ food habits were largely influenced by their nutrition literacy [18]. A cross-sectional survey reported a positive association between high nutrition literacy and desired vegetable and nut consumption [2]. 

A recent study reported a moderate level of nutrition literacy among Bangladeshi adults and evidence that socio-demographic factors were associated with nutrition literacy [19]. In Bangladesh, understanding how food choice is associated with an individual’s nutrition literacy and sociodemographic factors would be useful to effectively implement National Dietary Guidelines (2015), National Nutrition Policies (2015), and the Second National Plan of Action for Nutrition (2016–2025) and achieve nutrition targets [20,21]. This study highlights the association between nutrition literacy and healthy eating behaviors among Bangladeshi adults, which utilizes the data from the parent study, ‘Nutrition Literacy Study 2021′.

## 2. Materials and Methods

### 2.1. Research Design and Sample 

**Figure 1:**
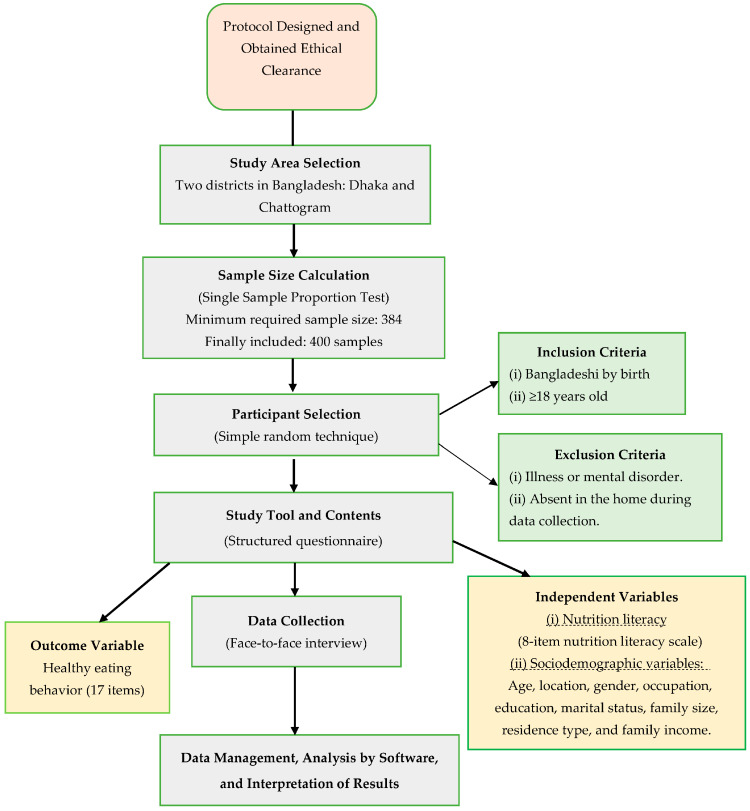
Methodological framework of the study.

This cross-sectional study identified the association between healthy eating behaviors and nutrition literacy in adults who live in the Dhaka and Chattogram districts of Bangladesh. Participant eligibility was based on the following criteria: (i) Bangladeshi citizen, (ii) being an adult (≥18 years), and (iii) living with no psychological disorders. A sample size of 384 was calculated using the single sample proportion test by assuming 50% of the expected prevalence of healthy eating behaviors among Bangladeshi adults, as there is no previous Bangladeshi study of this type. A 95% confidence interval and 5% of margin of error were also considered. In anticipation of missing data, and to obtain an optimal sample size, 400 adults were recruited for this study. Participants were randomly recruited from the selected districts (n = 2) with an equal allocation measure (i.e., 200 participants from each study district). The overall methodological framework of this study is depicted in Figure 1.

### 2.2. Study Variables 

The healthy eating behaviors of an individual (outcome variable) were assessed by asking whether people followed the National Dietary Guidelines in Bangladesh [22]. The guidelines contain a set of advisory statements providing principles and criteria for good dietary practices to achieve better health and well-being. It also suggests the use of healthy preparation and cooking methods for the retention of micronutrients in the food. The tool consists of 17 items evaluating individuals’ eating behaviors and nutrition-related practices. Each item, except item number 14 and 16, had three possible options: “Regular (Everyday per week)”, “Occasionally (at least three days per week)” and “Never”. Another item consisted of “Has your body weight been measured?” in which the responses were categorized as “Regular (at least once a month)”, “Occasionally (once in 2-3 months)” and “Never”. Finally, the item regarding health check-up was phrased as “Undertake clinical check-up?” in which the responses were categorized as “Regular (once in a year)”, “Occasionally (once in 2-3 years)” and “Never”. For assessing healthy eating behaviors, we assigned scores ranging from 0 to 2 (2, 1, and 0 points given for “regular”, “occasionally”, and “never”, respectively) for each of the items. The score of one item was reversed for ease of interpretation; “Eat foods containing excessive fats and oils/Eating fast foods”. A total score of an individual was computed by summing up all the scores of the 17 items (score range 0 to 34). Respondents having a higher healthy eating behavior score indicated a higher level of healthy eating. The internal consistency of this part was within the acceptable limits (Cronbach’s α = 0.71). 

Participants’ demographics and nutrition literacy were the explanatory (predictor) variables. Sociodemographic characteristics of the participants, including age, area of living, gender, occupation, education, marital status, family size, residence type, and monthly family income were obtained. Nutrition literacy was measured by a validated 8-item nutrition literacy scale, developed by Liao et al. [3]. Based on the definition of nutrition literacy [4,23], this scale included information on capacities regarding nutritional information in five domains: ‘obtain’ (2 items), ‘understand’ (2 items) ‘analyze’ (one item), ‘appraise’ (2 items) and ‘apply’ (one item). A point of 1 (very difficult) to 4 (very easy) was given to each participant’s response for the 8-items. A sum score was computed (range 8 to 32), a lower score indicates a lower nutrition literacy and vice-versa. The Cronbach’s alpha of this section of the questionnaire was 0.81 representing a good level of internal consistency. 

### 2.3. Ethical Statement 

Ethical clearance to conduct the study was obtained from the Departmental Ethical Committee of the Department of Food Microbiology, Patuakhali Science and Technology University, Bangladesh. The Helsinki Declaration of 1964 and its subsequent adjustments were followed in conducting the study. Respondents signed an informed consent form that explained the study’s purposes, possible risks, advantages, and confidentiality of their personal information. Moreover, participation was entirely up to them, and we provided them complete freedom to accept or decline. 

### 2.4. Data Collection Procedure

Data collection was carried out by six trained research assistants incorporating face-to-face interviews using a structured questionnaire that took place from May 2021 to September 2021. The research assistants were instructed by the principal investigator of the study through an online training session, demonstrating various components of the questionnaire, sampling techniques, and study eligibility requirements. The questionnaire was first prepared in English and then translated into Bengali (native language) for appropriate communication during data collection. The questionnaire was pre-tested among 20 adults to identify any confusing questions and to gain a better insight into the amount of time needed for the interview. Each interview took approximately 10-15 min and data from the pilot survey were not included in the current study results. 

### 2.5. Statistical Analyses 

Descriptive statistics (i.e., frequencies and percentages) were computed to observe the distribution of the variables. Since the outcome variable (healthy eating score) was continuous, the Pearson correlation was performed to determine the association between healthy eating behaviors and nutrition literacy. Linear regression models were fitted to observe the differences in healthy eating behavior associated with variation in nutrition literacy. Sociodemographic characteristics (such as location, age, gender, occupation, education, marital status, family monthly income, residence, and family size) were included in the model to observe the adjusted effect of nutrition literacy on healthy eating behavior. The variance inflation factor (VIF) was estimated to check the multicollinearity among covariates (mean = 1.47). Akaike information criterion was checked regarding the linear regression after fitting the models. The standardized beta coefficient (β) was considered to quantify associations. *p*-values less than 0.05 were considered statistically significant. Stata version 17.0 (StataCorp, College Station, TX, USA) was used to perform the data analysis.

## 3. Results 

The demographics of the participants are illustrated in Table 1. Of 400 participants, 61.5% were male and 58.3% were aged between 18 to 29 years, with a mean age of 30.3 (SD: ±8.7) years. One-third of the participants (33%) were students. About 63% had completed above higher secondary schooling. 

The mean score of healthy eating behavior was 21.8 (SD = 4.8, Range: 5–33) out of 34 points. This indicated a 64.1% (21.8/34.0 × 100) healthy eating score among the participants, indicating a moderate level of healthy eating practices. Responses of the participants to each of the healthy eating-related questions are summarized in Table 2.

Table 3 reports the unadjusted and adjusted linear regression models, which were used to identify the association between healthy eating behaviors and nutrition literacy. The unadjusted model revealed that nutrition literacy scores were positively associated with healthy eating behavior scores (β = 0.275, *p* < 0.001). After adjusting for demographics, the adjusted regression model showed a significant positive association between nutrition literacy scores and healthy eating behavior scores. The model also indicated that a unit increase in nutrition literacy equated to an increase in the mean healthy eating behavior score by 0.223 units (β = 0.223, *p* < 0.001).

**Figure 2:**
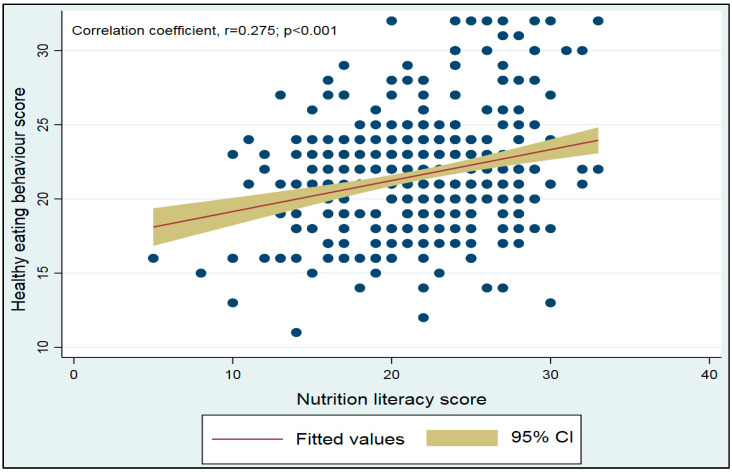
Scatter plot showing the correlation between healthy eating behavior scores and nutrition literacy scores based on the sample of data (N = 400).

Figure 2 represents the relationship between participants’ nutrition literacy scores and healthy eating behavior. As shown in Figure 2, a moderately positive relationship was found between nutrition literacy and healthy eating behavior of participants (r = 0.275, *p* < 0.01).

## 4. Discussion 

The purpose of this study was to investigate the association between healthy eating behaviors and nutrition literacy in a sample of Bangladeshi adults. Healthy eating behavior was associated with nutrition literacy in Bangladeshi adults. The finding is in-line with previous studies in various population groups including adults, students, and adolescents [2,18,24,25]. The findings favor the need for nutrition education for people in both rural and urban areas of Bangladesh. Few studies have found contrary results, for example, Natour et al. [26] found a minimal association between nutrition literacy and dietary behavior in Palestinians. A probable reason behind the weak correlation between nutrition literacy and dietary behavior, according to Natour et al. [26], could be people’s inability to understand nutrition-related messages and incorporate them into dietary practices. As part of the rapid demographic and epidemiological transition in the last few decades, most people in Bangladesh are shifting their eating patterns in general, resulting in a high prevalence of overweight or obesity, diabetes, and cardiovascular diseases [27,28]. This presents an urgent need to disseminate educationally appropriate nutrition-related information at virtual platforms and in-person settings to sensitize all population groups in Bangladesh.

Diet-related knowledge, attitudes, and behaviors are crucial in dietary health promotion at the personal level [29,30,31]. The Knowledge-Attitude-Behavior (KAB) model for nutrition education complies with the idea that an individual who is exposed to new information will pay attention to it, and gain new knowledge, leading to changes in attitude, which can ultimately shape healthy dietary practices [32]. However, “knowledge” must be motivational in nature, as specified by KAB models for changing attitudes and behavior. Healthy eating behavior depends on multiple factors. Previous research showed that nutrition literacy can be influenced by different factors. For example, Banna and colleagues [19] reported Bangladeshi adults’ nutrition literacy was associated with sociodemographic factors, such as residence, occupation, education level, family income, and personal beliefs (e.g., self-perceived need for access to nutrition-related information). Findings of the present research highlight the need to improve adults’ nutrition literacy by addressing demographic factors, which might impact positively attitudes, and subsequently improve healthy eating behaviors in Bangladesh. 

As mentioned earlier, healthy eating behavior relies on multiple factors and the interaction among these factors is complex. Therefore, it is difficult to explain the phenomenon using data collected through a study with a small sample size. In our case, the adjusted R^2^ is 0.354 which indicates moderate predictability of the regression model. Moreover, gender bias in household food preference/choice could also affect the healthy eating behavior score. Although there was no available information to observe how household food preference is associated with the gender of the family members in the study area, a report based on fieldwork in Barisal and Dinajpur mentioned that the decision of household food preference is equally contributed by both husbands and wives nowadays [33]. If so, a difference in the proportion of males and females in the sample (68.5% vs. 31.5%, respectively) might not overestimate or underestimate the estimated healthy eating behavior because the gender of the respondents was adjusted in the statistical model. Moreover, 58.3% of respondents were between 18-29 years of age, and their dietary behavior was likely to be highly influenced by their parents, considering Bangladeshi contexts. Furthermore, there was no information about how the family food preference, such as preference towards a particular food group, is affected by gender. Therefore, further studies are recommended to address these issues and examine causal pathways between nutrition literacy and healthy eating behaviors in Bangladeshi adults.

### Strengths and Limitations

The present study has some inherent limitations: causality cannot be claimed as the study design was cross-sectional and the sample size was not representative for Bangladesh as the study was limited to two regions in Bangladesh and was predominately young adults. We only considered the internal consistency of the items in the nutrition literacy scale by calculating Cronbach’s alpha and back-translation of the scale used for assessing the main variables of interest in this study. Since the Bengali version of the nutrition literacy scale has not yet been validated, a statistics-based adaptation and validation study for the Bangladeshi population is highly recommended. Another key consideration is that individuals with or without economic resources to follow healthy eating guidelines should be analyzed separately because the underlying relationship between knowledge and behavior has to be interpreted differently for the two groups. Moreover, social desirability biases and reporting biases from respondents could have occurred. For example, items to assess healthy eating behavior considered only three categories of responses, and there are some possibilities of in-between responses. In that case, for example, some respondents might be more likely to provide positive responses towards healthy eating which could lead to an overestimation of healthy eating behavior and vice-versa. Nonetheless, this study is one of the first to assess the association between nutrition literacy and eating behaviors in Bangladesh. Further, this study has rigorous methodological and analytical approaches.

## 5. Conclusions

Findings from this study illustrate a positive moderate relationship between nutrition literacy and healthy eating behavior in Bangladeshi adults. Further research is needed to determine a causal relationship that may help inform the necessity of educational interventions for Bangladeshi adults to assist with meeting national nutrition-related targets. Such interventions may include content related to healthy nutrition practices, including information about the importance of a balanced diet and incorporating healthy dietary habits, and could be disseminated via community durbars, mass media (e.g., TV, radio, newspaper, and social media platforms), and educational institutions and hospitals. These tactics may help Bangladeshi adults meet the objectives put forth by several nutrition-focused initiatives such as the Sustainable Development Goals.

## Figures and Tables

**Table 1:** Socio-demographic characteristics of study participants (N = 400).

Variables	Number	Percentage
**Location**		
Dhaka	200	50.0
Chattogram	200	50.0
**Gender**		
Male	246	61.5
Female	154	38.5
**Age age (in years)**		
18–29	233	58.3
30–39	105	26.3
40–49	41	10.3
50 or above	21	5.3
**Occupation**		
Student	132	33.0
Business	61	15.3
Unemployed	37	9.3
Private job	82	20.5
Housewife	36	9.0
Others ^#^	52	13.0
**Education level**		
Primary education	41	10.3
Secondary and higher secondary	105	26.3
Under graduation	89	22.3
Graduation	78	19.5
Masters or above	87	21.8
**Marital status**		
Single	227	56.8
Married	164	41.0
Divorced, separated or widowed	9	2.3
**Family size**		
≤5 members	291	72.8
>5 members	109	27.3
**Permanent residence**		
City area	299	74.8
Sub-urban	47	11.8
Rural area	54	13.5
**Monthly family income (BDT)**		
≤20,000	115	28.7
21,000–40,000	137	34.3
>40,000	148	37.0

^#^ Others including doctor, teacher, laborer, and retired. BDT = Bangladeshi Taka (1 USD = 86 BDT).

**Table 2:** Assessment of healthy eating behaviors among study participants (N = 400).

Eating Behavior and Related Issues	Frequency of Eating		
	Regular	Occasionally	Never
Eat a variety of food from 6–8 food groups of food pyramid	45 (11.3%)	195 (48.8%)	160 (40.0%)
Eat unpolished rice, wheat	191 (47.8%)	80 (20.0%)	129 (32.3%)
Eat citrus and Vit-A rich fruits	62 (15.5%)	153 (38.3%)	185 (46.3%)
Eat vegetables (leafy and non-leafy)	25 (6.3%)	144 (36.0%)	231 (57.8%)
Eat fish/meat	15 (3.8%)	124 (31.0%)	261 (65.3%)
Eat pulses and legumes	49 (12.3%)	124 (31.0%)	227 (56.8%)
Eat foods containing excessive fats and oils/ eating fast foods	67 (16.8%)	167 (41.8%)	166 (41.5%)
Eat sweetened foods	191 (47.8%)	151 (37.8%)	58 (14.5%)
Drink milk and milk-based products	157 (39.3%)	113 (28.2%)	130 (32.5%)
Eat fresh, well-prepared foods	35 (8.8%)	110 (27.5%)	255 (63.7%)
Avoid overeating	46 (11.5%)	136 (34.0%)	218 (54.5%)
Eat food with proper chewing	49 (12.3%)	91 (22.8%)	260 (65.0%)
Always wash hands before meals	12 (3.0%)	42 (10.5%)	346 (86.5%)
Has your body weight been measured monthly?	145 (36.3%)	106 (26.5%)	149 (37.3%)
Perform exercise	184 (46.0%)	99 (24.8%)	117 (29.3%)
Undertake clinical check-up at least once a year	78 (19.5%)	142 (35.5%)	180 (45.0%)
Take enough rest and sleep (8 h)	30 (7.5%)	109 (27.3%)	261 (65.3%)

**Table 3:** Linear regression model for healthy eating behaviors explained by nutrition literacy.

Variable	Unadjusted Model			Adjusted Model ^†^		
	β	95% CI	*p* Value	β	95% CI	*p* Value
Nutrition literacy	0.275	0.24, 0.49	<0.001	0.223	0.18, 0.41	<0.001
**Model fitness**		**R^2^**	**AIC**	**Mean VIF**	**Adjusted R^2^**	**AIC**
		0.076	2367.03	1.47	0.354	2231.91

Note: β indicates the standardized beta coefficient. CI implies to confidence interval. ^†^ The adjusted model of regression analysis was adjusted for location, age, gender, occupation, education, marital status, family monthly income, residence, and family size. AIC refers to Akaike information criterion and VIF implies to variance inflation factor.

## Data Availability

Data used in this analysis can be obtained by contacting the first author.
